# Relational autonomy in pediatric healthcare: A scoping review

**DOI:** 10.1177/09697330251366601

**Published:** 2025-09-12

**Authors:** Amarens Matthiesen, Nicole Drumm, Alison J Gerlach, Donna Koller, Tieghan Killackey

**Affiliations:** 1 7984 School of Early Childhood Studies, Faculty of Community Services, Toronto Metrpolitan University, Toronto, Ontario, Canada; 2 School of Nursing, York University, North York, Ontario, Canada; 3 8205 School of Child and Youth Care, Faculty of Education, University of Victoria, Victoria, British Columbia, Canada; 4 Lawrence Bloomberg Faculty of Nursing, University of Toronto, Toronto, Ontario, Canada; 5 Peter Munk Cardiac Centre, University Health Network, Toronto, Ontario, Canada

**Keywords:** Children, decision-making, healthcare, pediatric, relational autonomy, scoping review

## Abstract

**Background:**

The concept of relational autonomy has gained popularity for accentuating the social embeddedness of autonomy. In pediatrics, relational autonomy provides a framework for conceptualizing the complexities of healthcare processes, such as shared decision-making and children’s transitions to adult care systems. However, a lack of clarity exists regarding how to define and operationalize relational autonomy in pediatric healthcare. To our knowledge, no reviews of literature have been conducted to better understand this concept in pediatrics and guide further research.

**Research Aim:**

The purpose of this scoping review was to describe literature focusing on relational approaches to autonomy in the context of pediatric healthcare.

**Research design:**

A scoping review methodology proposed by Arksey and O’Malley and the Joanna Briggs Institute guided this review. Seven databases were searched for literature published between 2004 and 2024. Out of 2895 potentially relevant publications, a total of 28 articles were included for review.

**Ethical Considerations:**

This study was conducted and reported in accordance with recognized scientific scoping review guidelines.

**Findings:**

Various terms were used to describe relational approaches to autonomy in pediatrics. These terms shared a common goal of promoting a more holistic view of children and their decision-making processes in pediatric care. Most articles relied on adult perspectives (e.g., caregivers and healthcare providers) to shed light on shared decision-making processes. The perspectives of children and nurses were underrepresented in the literature, especially in the context of the Global South.

**Conclusion:**

Additional research is needed to capture the lived experiences of a wider range of individuals with developing and exercising autonomy using diverse research methods. As the perspectives of nurses and children remain excluded from literature, new knowledge on their experiences with autonomy can inform the delivery of care that is ethically and morally sound.

## Introduction

Children’s rights to have control over their bodies and participate in healthcare decision-making are widely recognized as important facets of ethical pediatric care.^1–4^ These rights promote a view of children as active citizens and agentic beings in relation to their health and healthcare.^5,6^ Children’s rights are underpinned by a recognition of children’s evolving autonomy—a concept associated with voluntary and rational choice.^5,6^

Dominant understandings of autonomy purport that one’s autonomy, self-determination, and decision-making capacities reside within a “rational” and morally “free” person.^2,6,7^ This assumption is rooted in Western liberal ideals of the “normal” adult subject, whose choices are only considered “autonomous” when they are uncoerced by contextual elements.^5^ In turn, the diverse ways in which personal circumstances, social relations, identities (e.g., gender, age, and ethnicity), and structural elements (e.g., ethnocentrism) can shape how autonomy is experienced and exercised become overlooked.^3^

Feminist theorists have critiqued traditional, individualistic conceptions of autonomy to reconceptualize this concept in relational terms.^2^ The construct of relational autonomy emerged to move beyond individualistic views of autonomy and capture the social embeddedness of persons and their identities, needs, interests, personhood, and autonomy.^6–8^ In feminist ethics scholarship,^6,7,9^ relational autonomy has been defined as an umbrella term to encapsulate the idea that:

*…persons are socially embedded, and agents’ identities are formed within the context of social relationships and shaped by a complex of intersecting social determinants, such as race, class, gender, and ethnicity.* ^6^

Despite the value of a relational outlook on autonomy in the context of pediatric healthcare, the application of this construct in pediatrics remains underexplored and inconsistently defined. This paper addresses this gap by mapping extant literature on relational understandings of autonomy in pediatric healthcare contexts.

## Background

Contemporary pediatric healthcare is characterized by increasing rates of complexity.^10,11^ For example, the rate of children diagnosed with medical complexities and chronic conditions is rising, which often necessitates frequent and lengthy hospital stays, and interprofessional collaboration over multiple healthcare sites.^10^ Alongside the rise of family-centered approaches to care, caregivers are also becoming more involved in the delivery of pediatric healthcare.^11^ Relational autonomy offers a valuable lens to account for these complexities by attending to the interconnectedness of children, caregivers, and healthcare providers in pediatrics. In addition, relational autonomy attends to other important dimensions of *relatedness*, including the physiological, psychological, emotional, and cognitive-developmental aspects of healthcare and decision-making.^1,12^

Relational autonomy holds particularly important implications for understanding children’s participation in healthcare decision-making. As children's decision-making rarely occurs in isolation, relational autonomy provides legitimacy for replacing the traditional, paternalistic dipole (physician-patient) with a triangle of actors (children/caregivers/healthcare providers) to promote a *shared* view of decision-making.^12–17^ Relational autonomy balances the voice of the child with the (at times, competing) needs, wishes, vulnerabilities, and interests of caregivers and providers in a holistic manner.^1,18–20^ This relational framing can promote a view in which children’s status is shared with adults as rights-holders to guide shared decision-making processes.^5^

Despite the value of relational autonomy in capturing pediatric healthcare complexities, its uptake in practice remains a challenging endeavor.^1^ One key reason for this may be that autonomy is a complex concept that is often used interchangeably with the notion of agency—also defined as the capacity for “choice.”^21,22^ Similarities and differences between autonomy and agency remain under-examined.^23^ While both autonomy and agency guide medical interventions and decision-making, delineating what they mean in different practice contexts and situations remains complex and confusing.^24^ Service providers and institutional contexts may promote varying ideas about how children’s autonomy may differ from that of adults.^1^ As childhood is a time period characterized by development and protectionism, children are often not considered as fully autonomous rights-holders.^5^ Healthcare providers must delineate between parental autonomy and the developing autonomy of a child.^1^

Adding to such conceptual challenges, autonomy is often equated with interrelated principles such as informed consent/assent, and children’s (evolving) capacity, and competence.^6^ For pediatric healthcare providers such as nurses, these principles represent ethical challenges that are complicated by surrogate decision-makers, children’s cognitive development, and children’s health status, to name a few.^1,22,24^ In light of these challenges, clear understandings of autonomy are needed to guide clinical practices that are morally and ethically sound and to realize children’s rights to protection, participation, and provision in pediatrics.^1,17,25^

## Research Aim and Rationale

While relational autonomy originated in adult care contexts,^26^ there has been a growing interest in linking relational autonomy to pediatric healthcare issues, including shared decision-making^12,14,15,17,18^ and palliative decision-making processes.^2,13,25,27^ Despite this growing interest, no previous literature reviews have explored how relational autonomy has been applied to pediatrics. Therefore, the aim of this scoping review was to identify and describe literature focusing on relational autonomy in the context of pediatrics, including nursing practice. The secondary aim was to explore how relational autonomy has been conceptualized across extant literature, including its theoretical underpinnings and characteristics.

The rationale for this review emerged through a collaboration between authors TK and AM and their clinical experiences as a registered nurse and certified child life specialist (pediatric psychosocial care provider), respectively. In clinical practice, the authors were struck by the challenges and nuances associated with fostering children and young people’s sense of independence and autonomy particularly in relation to decision-making^18,20^ and transitioning^28,29^ to adult care. Their clinical experiences, as well as a perceived lack of literature on this topic, strengthened authors’ interests in understanding how the concept of relational autonomy can meaningfully capture the complexities associated with autonomy in pediatric healthcare.

## Ethical statement

While this study did not require approval from an institutional ethical review board, it was conducted and reported in accordance with recognized scientific scoping review guidelines. In particular, the PRISMA Extension for Scoping Reviews (PRISMA-ScR) checklist was used to guide the review and the reporting of findings.

## Research Design

This scoping review was conducted to explore how the construct of relational autonomy has been applied to the context of pediatric healthcare across extant literature. A scoping review methodology was deemed as the most suitable approach to explore the breadth and depth of the literature^30^ as: (a) no previous reviews have been conducted on this topic, and (b) relational autonomy is a concept that has been applied to a broad range of healthcare and academic disciplines.

The literature search was guided by a methodological framework proposed by Arksey and O’Malley^32^ and further refined by the Joanna Briggs Institute (JBI).^30^ The scoping review findings are reported using the PRISMA extension for Scoping Reviews (PRISMA-ScR).^30^ The JBI methodology outlines that scoping reviews pay attention to describing the planned approach to evidence searching, selection, data extraction and presentation of the evidence prior to conducting the review.^31^ A study protocol was published in the Open Science Framework repository which can be accessed via: https://osf.io/u2yqv/.

In line with guidelines published by Arksey and O’Malley,^32^ this scoping review was conducted following five methodological stages: (1) identification of the research question, (2) identifying relevant studies, (3) selection of studies, (4) charting the data, and (5) collating, summarizing, and reporting the results.

### Stage 1: Identifying the research questions

This stage included formulating a series of overarching objectives to guide the study. Following an initial, broad literature search on the notion of relational autonomy, the following research questions were developed to guide the review:

○ How has relational autonomy been defined in the context of pediatric healthcare?
○ What is known about relational autonomy in pediatrics and within specific healthcare disciplines?
○ What are the implications of adopting a relational autonomy approach for clinical practice, including nursing?

### Stage 2: Searching for relevant studies

This stage was initiated by searching seven electronic databases: CINAHL (EBSCOhost), ERIC, Sociology Collection (ProQuest), APA PsycInfo (EBSCOhost), MEDLINE (OVID), Web of Science, and Scopus. Reference lists of pertinent articles were also manually searched for additional articles. The literature search strategy was developed in consultation with a university librarian at the University of Victoria with expertise in conducting scoping and systematic literature reviews.

Combinations of keywords and index terms that focus on the population of interest (e.g., children, youth, and pediatrics) and concept of interest (e.g., relational autonomy, relational approach, and relational care) were entered into the databases using Boolean operators. Search terms pertaining to relational autonomy were expanded to include related terms that signify a relational approach to conceptualizing autonomy, such as “relational capacity” or “shared autonomy,” to maximize search results and ensure all relevant articles were captured. The search was completed in November 2024. Table 1 depicts an example overview of the key terms entered in one of the databases, MEDLINE.

**Table 1.** Search strategy overview – Key terms entered in MEDLINE.

#	Category	Query
1	Relational autonomy	(“Relational autonomy” or “relational approach*” or “shared autonomy” or “relational care” or “relational personhood” or “relational person-hood” or “relational anthropology” or “relational account” or “relational capacit*” or “relational health” or “personal autonomy” or “patient* autonomy” or “beyond autonomy” or “beyond individualism” or “relational turn” or “relational interest*”).tw,kf
2	Relational autonomy	Personal autonomy/ or relational autonomy/
3	Population	((Child* or youth or adolescen* or teen* or pediatric* or paediatric* or toddler* or infan* or neonate* or newborn* or “new-born*”) adj5 (care* or health* or medic* or wellness* or wellbeing or “well-being”)).tw,kf
4	Context	Adolescent health/ or adolescent health services/ or child health/ or child health services/ or maternal health services/
5	Context	(Pediatric* or paediatric*).tw,kf
6	Context	Exp pediatrics/
7		1 or 2
8		3 or 4 or 5 or 6
9		7 and 8

### Stage 3: Study selection

The selection of relevant articles commenced by defining the inclusion and exclusion criteria. The inclusion criteria are displayed below: (Table 2).

**Table 2.** Study inclusion and exclusion criteria.

Criteria	Details
Inclusion criteria	• Article published in peer-reviewed journal (including empirical studies and commentaries)
	• Focuses on relational approach (es) to understanding autonomy in pediatric healthcare
	• Focuses on children and youth ages 0–24 years (including the perinatal period)
	• Published in the last 20 years (2002–2024) in English
Exclusion criteria	• Places a focus on non-pediatric healthcare-related contexts (e.g., education)
	• Places insufficient focus on relational approach (es) to autonomy (e.g., approach does not form part of article objective(s) and/or is only described in findings)
	• Literature type includes letters, book reviews, review articles, and unpublished papers

The search was limited to the last 20 years as the early 2000s saw increased attention to relational autonomy within pediatric health and bioethics literature.^6,8^ This date limit therefore ensured that any included literature remained relevant to current healthcare contexts and practices. With regards to the age limit (0–24 years), the authors initially focused the literature search on children and youth aged between zero and 21 years, as this is a commonly used reference range in pediatrics.^33^ However, the authors expanded this age range as an initial broad scan of the literature pointed to a focus on youth up to 24 years of age. An age limit of 24 years allowed for a broad array of literature to be captured, including potential articles that capture children’s transitions from pediatric to adult care spanning the ages 12–25 and associated implications for understanding autonomy.^29^

**Figure 1.**
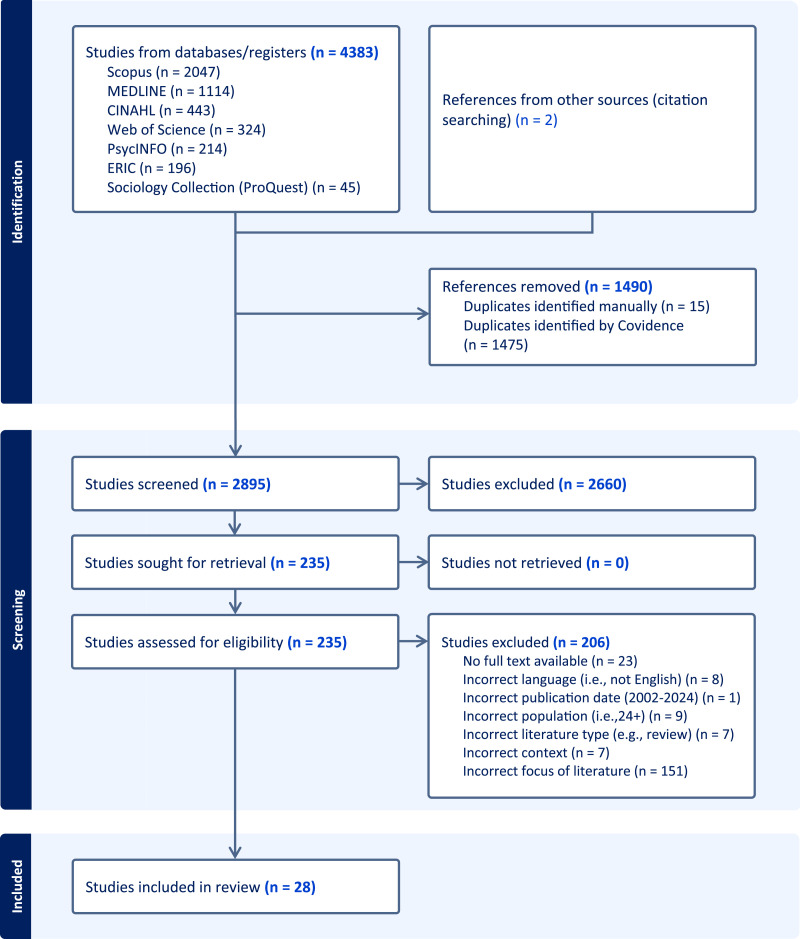
Flow chart of the study selection procedure.

After the identification of pertinent literature, two reviewers (AM and ND) independently and concurrently screened titles and abstracts of 2895 articles using Covidence software (Veritas Health Innovation). Covidence is a web-based collaboration software platform that streamlines the production of systematic and other literature reviews and automatically removes duplicate entries. Following this screening stage, the full texts of 235 articles were carefully reviewed, of which 28 articles met all inclusion criteria. Any decisional conflicts or ambiguities in article selection were resolved by consensus through discussion with a third reviewer (TK). Figure 1 presents an overview of the literature search process.

### Stage 4: Charting the data

Charting the data involved synthesizing and interpreting key items of information obtained by the included articles in qualitative form.^32^ This stage commenced by developing a data extraction form in Microsoft Excel. In this data charting form, two authors AM and ND extracted pertinent information from the sample of included articles. Information extracted from the articles were categorized according to the following variables:

• Author(s) and year of publication
• Article type (e.g., research or discussion/commentary paper)
• Geographical context
• Population type (i.e., children, caregivers, and/or healthcare providers)
• Article objective(s)
• Methodology
• Definitions and described purpose(s) of relational autonomy approach
• Healthcare discipline context(s)
• Health condition(s) focus
• Key findings
• Implications for future research

### Stage 5: Collating, summarizing, and reporting the results

To explore how the concept of relational autonomy has been conceptualized and applied to pediatric healthcare, the extracted data from the 28 included articles were analyzed through a thematic approach.^34^ Consensus on overarching themes and sub-themes was established by collaborative discussions between team members. 

## Findings

The literature search yielded 235 relevant citations, of which 28 articles were included for analysis in this scoping review. An overview of all included articles is presented in Table 3. Collaborative analysis identified several key themes in the data, including (1) Varying conceptualizations of relational autonomy, (2) Relational autonomy in shared decision-making processes, (3) Adult-dominated perspectives on relational autonomy, and (4) Implications for clinical practice. Before discussing the themes in more detail, a summary of the key descriptive characteristics of the literature is presented in Table 4.

**Table 3.** Overview of included articles.

#	Author(s) and Year	Article title	Journal
1.	Akre et al. (2014)^51^	From controlling to letting go: What are the psychosocial needs of parents of adolescents with a chronic illness?	Health Education Research
2.	Atkins (2023)^49^	What matters morally for children receiving health care	Child Heath Care
3.	Bogossian et al. (2020)^39^	Contextualized autonomy in transitional care for youth with neurologic conditions: The role of the pediatric neurologist	Journal of Child Neurology
4.	Brisson et al. (2024)^26^	A mixed-methods study exploring Colombian adolescents’ access to sexual and reproductive health services: The need for a relational autonomy approach	Journal of Bioethical Inquiry
5.	Buchbinder (2009)^41^	The management of autonomy in adolescent diabetes: A case study of triadic medical interaction	Health
6.	Donetto & Maben (2015)^37^	“These places are like a godsend”: A qualitative analysis of parents' experiences of health visiting outside the home and of children's centres services.	Health Expectations
7.	Fay et al. (2021)^27^	The relationship between proxy agency and the medical decisions concerning pediatric patients in palliative care: A qualitative study.	BMC Palliative Care
8.	Fernandes et al. (2022)^42^	Communicating the spinal muscular atrophy diagnosis to children and the principle of autonomy	BMC Pediatrics
9.	Gastmans et al. (2013)^36^	From birth to death? A personalist approach to end-of-life care of severely ill newborns	BMC Medical Ethics
10.	Ganya et al. (2016)^45^	Autonomy of the child in the South African context: Is a 12-year-old of sufficient maturity to consent to medical treatment?	BMC Medical Ethics
11.	Gunning et al. (2022)^38^	Clinician views on and ethics priorities for authorizing medical cannabis in the care of children and youth in Canada: A qualitative study	Canadian Medical Association Open Access Journal
12.	Hanna et al. (2013)^44^	Parent–adolescent dyads: Association of parental autonomy support and parent–adolescent shared diabetes care responsibility	Child: Care, Health and Development
13.	Navne & Svendsen (2018)^47^	A clinical careography: Steering life-and-death decisions through care	Pediatrics
14.	Paron (2024)^24^	Exploring child-patient autonomy: Findings from an ethnographic study of clinic visits by children	Child Indicators Research
15.	Paron & Kutsar (2014)^40^	Creation of child-patient’s autonomy in a child-parent-doctor relationship: Medical doctors’ perspectives	Childhood
16.	Partridge (2014)^14^	Adolescent pediatric decision-making: A critical reconsideration in the light of the data	HEC Forum
17.	Pinto (2004)^43^	Intersections of gender and age in health care: Adapting autonomy and confidentiality for the adolescent girl	Qualitative Health Research
18.	Racine et al. (2013)^50^	Respect for autonomy in the healthcare context: Observations from a qualitative study of young adults with cerebral palsy	Child: Care, Health and Development
19.	Roden & Ryan (2023)^35^	Privacy, parental consent, and relational autonomy	Journal of Adolescent Health
20.	Ruhe et al. (2016)^17^	Relational capacity: Broadening the notion of decision-making capacity in pediatric healthcare	Journal of Bioethical Inquiry
21.	Salter (2013)^61^	Should we tell Annie? Preparing for death at the intersection of parental authority and adolescent autonomy	Narrative Inquiry in Bioethics
22.	Salter (2014)^12^	Resisting the siren call of individualism in pediatric decision-making and the role of relational interests	Journal of Medicine and Philosophy
23.	Sawyer & Rosenberg (2020)^15^	How should adolescent health decision-making authority be shared?	AMA Journal of Ethics
24.	Silber (2011)^52^	Treatment of anorexia nervosa against the patient's will: Ethical considerations	Adolescent Medicine: State of the Art Reviews
25.	Szabat (2024)^25^	Parental agency in pediatric palliative care	Nursing Inquiry
26.	Walter & Ross (2014)^1^	Relational autonomy: Moving beyond the limits of isolated individualism	Pediatrics
27.	Yates (2011)^46^	Ethics for the pediatrician: Religion and spirituality in pediatrics	Pediatrics in Review
28.	Zielinksi et al. (2020)^13^	Relational decision‐making in the context of life‐limiting fetal anomalies: Two cases of anencephaly diagnosis	Journal of Midwifery & Women's Health

**Table 4.** Key characteristics of included articles.

Characteristic	No (%)
Article type	
Research paper	16 (57.1)
Qualitative	13 (81.3)
Quantitative	1 (6.3)
Mixed methods	2 (12.5)
Discussion/Theoretical paper	8 (28.6)
Commentary/Editorial	4 (14.3)
Publication year	
2004–2014	13 (46.4)
2014–2024	15 (53.5)
Location of corresponding author	
United States	12 (42.8)
Canada	3 (10.7)
Switzerland	2 (7.1)
Estonia	2 (7.1)
Other	9 (32.1)
Discipline	
Pediatrics (general)	15 (53.5)
Bioethics	5 (17.9)
Adolescent medicine (including sexual and reproductive health services)	4 (14.3)
Palliative care	2 (7.1)
Neurology	1 (3.6)
Perinatal care	1 (3.6)
Health condition focus	
Not applicable	18 (64.3)
Chronic conditions	8 (28.6)
Fetal anomalies (anencephaly)	1 (7.1)
Mental health conditions (anorexia nervosa)	1 (7.1)
Patient population age	
Children (ages 2–12)	10 (35.7)
Adolescents (age 13–19)	10 (35.7)
Unspecified	4 (14.3)
Young adults (ages 20–24)	2 (3.6)
Young children (ages 0–2)	2 (3.6)

### Descriptive characteristics of included articles

Most articles were empirical research papers (*n* = 16), which employed qualitative (*n* = 13), mixed methods (*n* = 2) or quantitative (*n* = 1) methodological approaches. Other article types included discussion/theoretical papers (*n* = 8) and commentaries/editorials (*n* = 4). The majority of included articles were published in 2014 (*n* = 4), 2020 (*n* = 3), and 2023 (*n* = 5).

In terms of geographical location (of the corresponding author), included articles represented a total of 13 countries. Most (*n* = 26) articles were published in the context of the Global North, including the United States (*n* = 12), followed by Canada (*n* = 3), Switzerland (*n* = 2), Estonia

(*n* = 2), Australia (*n* = 1), Belgium (*n* = 1), Denmark (*n* = 1), Mexico (*n* = 1), Poland (*n* = 1), Portugal (*n* = 1) and the United Kingdom (*n* = 1). Solely two articles represented the Global South, specifically Colombia (*n* = 1) and South Africa (*n* = 1).

Relational autonomy was also applied to various healthcare disciplines, including bioethics (*n* = 5), adolescent medicine (including sexual and reproductive health services) (*n* = 4), palliative care (*n* = 2), neurology (*n* = 1), and perinatal care (*n* = 1). Remaining articles (*n* = 15) focused on pediatrics in general. Articles which focused on specific health conditions included chronic health conditions (*n* = 8), such as (cerebral palsy) (*n* = 1), spinal muscular atrophy (*n* = 1), epilepsy (*n* = 1), and trisomy 13 (*n* = 1). In addition to chronic conditions, other articles focused on fetal anomalies (anencephaly) (*n* = 1) and mental health conditions (anorexia nervosa) (*n* = 1). Remaining papers (*n* = 4) concentrated on chronic conditions generally.

In terms of children’s age, the majority of articles pertained to children aged between two and 12 years (*n* = 10), followed by adolescents aged between 13 and 19 (*n* = 10). Some articles also focused on young adults (*n* = 2) and young children aged between zero and two years (*n* = 2). The remaining four articles did not specify an age range.

### Varying conceptualizations of relational autonomy

A predominant finding pertains to the ways in which the included literature reflected rich variations in conceptualizations of relational approaches to autonomy. Only three articles.^1,26,35^ (11%) used the term “relational autonomy” in the article title, and five articles^17,25,36–38^ (18%) embedded relational autonomy in their research objectives or discussions. For example, relational autonomy was used in a seminal discussion paper to critique individualistic views of autonomy in pediatrics.^1^ Another framed adolescents’ access to sexual and reproductive health services through a relational autonomy approach.^26^ Similarly, other authors explored the value of relational autonomy in the context of privacy concerns and parental consent in adolescent health.^35^ Overall, definitions of relational autonomy were broadly rooted in the principles of social relationships, embeddedness, and contextuality. Examples of how relational autonomy was defined are included in Table 5.

**Table 5.** Examples of definitions of relational autonomy.

Definition of Relational Autonomy	Source
“Relational autonomy perspectives are premised on a shared conviction, the conviction that persons are socially embedded and that agents’ identities are formed within the context of social relationships and shaped by a complex of intersecting social determinants, such as race, class, gender, and ethnicity.”	Walter & Ross (2014)
“Inspired by the value of relational autonomy, parents might view themselves as being embedded in relationships of support with individuals who share the weight of a difficult decision openly. The health care team should act in a way that supports the parents by inspiring trust in them and fostering the parents’ own self-trust and autonomy.”	Gastmans et al. (2013)
“A relational autonomy approach sees individuals as rooted within social networks [and] better conceptualizes the different ways that Colombian adolescents (and others) may wish to access sexual and reproductive health services.”	Brisson et al. (2024)

The remaining 16 articles (57%) utilized other, related terms that alluded to relational conceptualizations of autonomy (see Table 6). For example, articles used the terms “relational capacity”^17^ or “relational interests”^12^ to understand how pediatric decision-making is situated within and co-constructed by relationships. “Contextualized autonomy” was used to explore transitional care for youth with neurologic conditions.^39^ Other authors more indirectly situated the autonomy of children or adolescents within “trinomial” relationships with caregivers and healthcare providers^40–42^ or within the intersection of gender and age,^43^ for example.

**Table 6.** Variations in relational conceptualizations of autonomy reflected in the literature.

Associated Terms	Source(s)
Relational autonomy	Brisson et al. (2024); Roden & Ryan (2023); Walter & Ross (2014)
Child-patient autonomy	Paron (2024); Paron & Kutsar (2014)
Contextualized autonomy	Bogossian et al. (2020)
Proxy agency	Fay et al. (2021)
Parental autonomy	Hanna et al. (2013)
Relational capacity	Ruhe et al. (2016)
Relational interests	Salter (2014)
Parental agency	Szabat (2024)
Relational decision-making	Zielinski et al. (2020)

The terms “proxy agency” and “relational agency” were also employed to consider how children, caregivers, and healthcare providers share decision-making in the context of pediatric palliative care.^27^ The authors of this latter article also emphasize the close theoretical links between the concepts of autonomy and agency, and the challenges these concepts pose when they are applied to underage individuals.^27^ In addition, few articles focused on the role of caregivers in shaping children’s autonomy by employing terms such as “parental agency”^25^ or “parental autonomy.”^44^ Such “parental” outlooks pertained to child-caregivers dyads in shaping children’s health management and care.^44^

While varying conceptualizations of relational approaches to autonomy were evident in the literature, all included literature broadly shared a view of autonomy as being shaped by interrelated, contextual factors across different healthcare disciplines. Relational autonomy was described as promoting an awareness of how contextual factors, including interpersonal relationships, religion, spirituality, and culture^26,45^ can shape one’s exercise of autonomy.^39,46^ Pinto (2004) examined the intersections of gender and age in adolescent healthcare, while Walter and Ross (2014) discussed race, class, gender, and ethnicity as intersecting determinants in shaping autonomy.^1,43^

#### Theoretical underpinnings

Most articles (64%) did not explicitly elaborate on specific theoretical or philosophical underpinnings that shaped their conceptualizations of relational autonomy. In most cases, authors briefly defined how relational autonomy can be conceptualized without delving into its theoretical or philosophical foundations or definitions. Authors of ten articles clarified that they drew on (feminist) bioethics and sociological frameworks, principles, or perspectives. For example, three papers drew on bioethical scholarship to challenge individualistic and paternalistic ontologies in the context of healthcare, particularly in terms of decision-making processes.^12,13,47^

Six other papers (21%) rooted their discussions in related feminist (bio)ethics literature to frame a relational approach to personhood and autonomy,^28,49^ propose a relational (re)conceptualization of the notion of “capacity” in decision-making,^17^ critique the limits of individualist views of children’s autonomy,^1^ frame the principle of parental agency pediatric palliative care,^25^ and explore how caregivers experience health visiting services for their child.^37^ These papers specifically cited critical feminist scholars Mackenzie and Stoljar^6,48^ to frame how relational perspectives are premised on a “shared conviction” that individuals are socially embedded and that their identities are formed within social relationships. One other paper rooted its discussion of “child-patient” autonomy in relational sociological literature to consider the interconnectedness of individuals with society, in which “society is made *by* individuals but is not made *of* individuals.”^24^

#### Relational autonomy in shared decision-making processes

More than half (*n* = 19) (73%) of included articles discussed the application of relational autonomy in the context of healthcare decision-making processes.^1,12–15,17,24–27,40,41,43,45–47,49,50^ These decision-making processes were described as occurring in triadic relationships between children, caregivers, and healthcare providers. Healthcare providers were regarded as having an ethical responsibility to partner with patients to acknowledge their socio-emotional situation in informing healthcare decision-making.^13,17,41^

Healthcare “decisions” related to children’s capacity to consent to medical treatments,^17,45^ including decisions pertaining to life-limiting fetal anomalies,^13^ and generic “medical choices” in “routine” healthcare,^14^ for example. In the context of Africa, authors of one paper described a wider, community-based approach to children’s decision-making and framed children as being embedded in “communitarianism.”^45^ The authors proposed that, in traditional African thought, a person exists as “an extended entity embedded within a communal matrix of interrelations and interdependencies.”^45^ In turn, these authors suggested that decisions concerning the child, such as consenting to medical treatment, are discussed and determined by the community to which the child belongs.

### Adult-dominated perspectives on relational autonomy

Despite a predominant emphasis on “shared” decision-making in pediatric care with children, the foci of discussions regarding relational autonomy were primarily explored from the perspectives of (adult) healthcare providers. A total of six (21%) empirical studies focused on the perspectives and experiences of caregivers and/or healthcare providers.^37,38,40,42,47,51^ For example, one study explored how caregivers of adolescents with a chronic illness manage their child’s autonomy acquisition,^51^ while another explored how caregivers of a child with muscular atrophy experience receiving this medical diagnosis.^42^ Three studies interviewed or surveyed the perspectives of providers to gain insight into their views on triadic communication relationships or clinical decision-making issues.^38,40,47^ While two studies focused on the perspectives of physicians,^38,40^ one study interviewed physicians and nurses.^47^ In addition, Buchbinder (2009) conducted a case analysis of a single interaction between a child with diabetes, a nurse practitioner, and the child’s mother.^41^

Given the adult-focused nature of these articles, children’s perspectives on their experiences of developing and exercising autonomy were lacking. Among the 16 articles that represented empirical research studies, solely two (7%) articles used interviewing to garner first-hand insights from children and young people. These papers explored the perspectives of children with cerebral palsy regarding their autonomy^50^ and adolescents regarding sexual and reproductive health services.^26^

Two additional authors employed observation to explore children’s autonomy in the context of clinic visits^40^ and palliative care.^27^ Three other studies employed descriptive case study methodologies to shed light on the experiences of adolescents with ulcerative colitis,^43^ anorexia nervosa,^52^ and diabetes^41^ regarding their illness narratives, ethics of treatment, and triadic relationships with caregivers and providers, respectively.

#### Contrasting perspectives on children’s age

In the context of adult-dominated outlooks on relational autonomy, several articles pointed to contrasting ideas about the weight of children’s roles in “shared” decision-making approaches and responsibilities regarding care. Children’s role in shared decision-making was often undermined or omitted, primarily due to developmentalist concerns about children’s age and associated decision-making capacities. Instead, the role of caregivers was heightened to provide a supportive and, at times, substitutive role in shared decision-making processes.

For example, Paron et al. (2023)^40^ stated that the amount of involvement shared between a parent and a child in decision-making depends mainly on the child’s age. Another author^14^ argued that in pediatric care, adolescents should be treated as “apprentice decision-makers” who require the support of caregivers until they reach “full maturity” (at 18 years of age). Similarly, other authors promoted the idea of sharing the responsibility for care with caregivers, for example, in the context of diabetes^44^ and sexual and reproductive health.^26^ In one paper, younger adolescents were described as being more likely to involve caregivers in decision-making compared to older adolescents.^26^

In contrast to age-based outlooks on children’s decision-making capacities, three articles (11%) emphasized the role of “cultural” elements in shaping children’s autonomy in healthcare decision-making.^39,41,45^ For example, Ganya et al. (2016) that in South Africa, as a culturally diverse country, the western liberal notion of autonomy may not reflect the moral worldviews of children and caregivers. Thus, the authors proposed a move away from a general legal age of consent to a more relational, context-specific approach to determining the maturity of children in decision-making.

### Implications for clinical practice

#### Fostering a holistic view of pediatric care and decision-making

The overarching value of promoting a relational outlook on autonomy in clinical practice related to fostering holistic approaches to pediatric care and shared decision-making processes. This holistic perspective considers the social, emotional, and socio-cultural (including religious) dimensions of autonomy and recognizes the multiple actors involved in shaping autonomy.^12,47^ All included articles also promoted a holistic perspective to pediatric care, and primarily decision-making, by involving caregivers, providers, and other “contextual” elements in these processes. For example, Navne et al. (2018) emphasized that a relational framing of healthcare decision-making in Neonatal Intensive Care Units (NICU) represented a “new vocabulary” to help illuminate the moral and emotional components involved in healthcare decision-making.^47^

#### Moving beyond age-based understandings of children’s decision-making capacities

In the context of promoting more “holistic” accounts of care, five articles^12,15,17,26,45^ (18%) emphasized that the key implication of relational framings of autonomy pertains to moving beyond age-based understandings of children’s involvements in decision-making. For example, Salter (2014) suggested that the notion “relational interests” offers a comprehensive and holistic way to conceptualize healthcare decision-making compared to current age-based standards that guide practice in the United States.^12^ Similarly, Ganya et al. (2016) proposed that more context-specific approaches rooted in relational autonomy, rather than age-based consent laws, can better support South African children in consenting to medical treatments.^45^ In a Columbian context, Brisson et al. (2024) emphasized that adolescents valued more relationally embedded decision-making processes for sexual and reproductive health services.^26^ This subset of literature suggested than a relational autonomy approach can better respond to the needs and preferences of children and youth, rather than strict age-based outlooks on children’s decision-making “capacities.”

#### Informing healthcare policy developments

An additional implication of relational framings of autonomy in pediatrics pertained to their value in informing clinical policy developments, as suggested by five articles (18%).^14,26,37,40,50^ For example, Brisson et al. (2024) argued that a nuanced relational autonomy approach can inform policy developments that better align with the myriad of preferences that adolescents may have regarding their access to sexual and reproductive health services.^26^ Partridge (2014) suggested that a relational framing of decision-making can inform policy developments to ensure caregivers are routinely included in adolescents’ decision-making processes.^14^ Similarly, Racine et al. (2013) proposed that a relational framing of shared decision-making and its recognition that family interests can be integral in reinforcing the value of family-centered care policies.^50^ Relational framings of children’s autonomy were also suggested to improve “child-friendly” healthcare policy^40^ and inform policy developments on how pediatric community-based health services can promote parental autonomy.^37^

#### Outcomes for children and caregivers

As a final implication, four articles (14%) associated relational autonomy with enhanced outcomes for children and families in pediatrics. These outcomes included improved patient and caregiver satisfaction,^40,42^ patient adherence to treatment plans,^24^ increased caregiver resilience and confidence,^43^ and enhancing the alignment of care with the best interests of the child and care, as well as family values, and traditions.^46,47^ Promoting the idea of parental autonomy was also associated with enhanced “psychosocial outcomes” for adolescents with diabetes.^44^

## Discussion

The purpose of this scoping review was to map existing literature regarding relational autonomy in pediatric care to elicit an overall picture of literature in this field. The authors were interested in how relational approaches to autonomy have been applied to pediatric care, including how the theoretical underpinnings of relational autonomy have been described. Findings point to diverse conceptualizations of relational autonomy, which align with the understanding that relational autonomy does not refer to a single, unified conception of autonomy. Rather, relational autonomy represents an umbrella term for approaches to autonomy that are supported or constrained by social relationships and dynamics.^48^ Relational autonomy was most often applied at the “micro” level pertaining to interpersonal relationships between children, caregivers, and healthcare providers. Few authors^1,26,43,45^ discussed the intersection of social determinants of health in shaping autonomy (e.g., race, class, gender, and ethnicity). The literature reflected limited engagement with broader, “macro” power structures to frame how autonomy is understood and enacted.^3^ Included articles did not fully encapsulate the scope of relational autonomy as defined in foundational scholarship, which considers micro- and macro-level dimensions to be inextricably linked in shaping autonomy.^6^ This oversight may diminish the transformative potential of relational autonomy to interrogate how structural forces and inequities can influence children’s autonomy. Future research could extend the scope of relational autonomy by attending more closely to both micro- and macro-level dynamics in pediatric care.

In addition to varying (micro-level) definitions of relational autonomy, this review found limited descriptions of the theories that underpinned authors’ descriptions of relational autonomy. The lack of conceptual clarity regarding relational autonomy may contribute to the limited uptake of RA in clinical practice.^1,7^ Indeed, others have questioned whether relational autonomy compels in the abstract but fails the test of practice.^7^ While nuanced literature on relational autonomy exists, practical clarity has not emerged from theoretical discussions and they have had limited impact on practice.^1,7^ More empirical work is needed to close the gap between theory and practice, and strengthen the integration of relational autonomy in pediatrics.^7^

Despite this theory-practice gap, this review revealed that articles placed a clear emphasis on the value of relational autonomy in healthcare decision-making. Associating relational autonomy with shared decision-making reinforces the idea that children’s capacities for exercising autonomy are formed in relations with others.^7^ However, by expanding the number of individuals involved in decision-making, some have expressed caution about whether relational autonomy in fact “removes” the child from the center of decision-making and diminishes their right to decision-making.^7,53^ Conversely, the involvement of caregivers and healthcare providers may be viewed as facilitating and encouraging the child’s participation in decision-making, which has been linked to better health outcomes for children.^54^ The literature’s overarching emphasis on decision-making serves as a reminder that relational autonomy is valuable in framing other healthcare-related processes, such as advance care planning^20^ and transitioning to adult care.^39^ Thus, it may be valuable for future research to expand the application of relational autonomy beyond decision-making in pediatrics.

An additional implication of the study findings pertains to the perceived lack of attention paid to children’s perspectives on exercising their developing autonomy in pediatrics. Authors mainly focused on the perspectives of caregivers or healthcare providers (predominantly physicians). Considering that childhood is a crucial time for the development of skills and autonomy, the absence of children’s voices points to a gap in research. To reduce inequities in children’s autonomy, additional research is warranted to attend to children’s lived experiences with exercising their evolving sense of autonomy using creative, child-oriented methods.^23^

Moreover, study findings bring attention to the risks associated with age-based, developmentalist framings of children’s autonomy. Such binary perspectives frame children as either capable or incapable of making decisions based on arbitrary thresholds of age and social maturity.^23,55,56^ This framing can be particularly exclusionary for young children and children with disabilities.^54,55^ Such binary conceptions also stand in direct opposition to the premise of relational autonomy, which characterizes children’s development, decision-making, and associated autonomy as contextual, dynamic, and reciprocal. A relational lens reconceptualizes children’s development in terms of diversities, rather than (age-based) maturity.^23^ Even young children could then be attributed with decisional autonomy and they could benefit from having others (e.g., caregivers and providers) involved in the promotion of their interests.^21^ However, such transactional conceptions of children’s development are largely absent from literature.^25^ Future relational autonomy-related initiatives could therefore promote relational outlooks on children’s development and discuss the implications for understanding children’s autonomy.

## Implications for nursing practice

This review holds unique implications for nursing practice. The absence of nursing perspectives in discussions of relational autonomy reveals a gap in knowledge regarding how pediatric nurses perceive, experience, and conceptualize the development and exercise of autonomy in clinical care.^41^ As the majority of relational autonomy-related literature has been published in the context of adult nursing care,^57,58^ there is a need to further explore how relational autonomy may add value to pediatric nursing practice.^24^ In particular, additional research could shed light on the extent to which relational autonomy can respond to the moral responsibilities and concerns of nurses in their relationships with children and families.^59^ This area of focus aligns with growing literature on feminist ethics which has influenced the evolution of nursing ethics over the past 30 years.^59^ Relational autonomy can offer alternative ways of framing ethical issues, such as the role of pediatric nurses in informed consent procedures, shared decision-making, and balancing parental involvement and child responsibilities in care.^7,41^ A relational outlook on these issues could reveal the “messy gray zones” in pediatric nursing practice in which patients, families, and nurses think and act interdependently, emotionally, and, at times, conflictedly.^7^

Moreover, new knowledge on relational autonomy in pediatric nursing could reveal how nurses exercise their own sense of autonomy, and which elements may constrain or facilitate their autonomy.^41^ Supporting the autonomy of patients, as well as safeguarding nurses’ own autonomy, are core aspects of nursing care that can enhance the quality of care and patient safety.^59^ High levels of self-perceived autonomy among nurses has been associated with enhanced job satisfaction, cooperation with physicians, and lower job-related stress.^58^ Thus, additional research on relational autonomy in this context can shed light on the interactional dynamics of nurses’ healthcare encounters, with implications for patients and providers alike.

However, it must be recognized that the structural realities of pediatric healthcare settings can constrain the practical enactment of relational autonomy.^60^ Nurses often work under significant time pressures and institutional demands for efficiency.^60^ These systemic conditions can limit opportunities for nurses to engage in the time-intensive, relational work that is inherent to relational autonomy. These tensions must be accounted for when attempting to understand how relational autonomy can be realistically implemented and woven into nursing practice.

## Strengths and limitations

A strength of this review pertains to the sourcing of literature from multiple relevant databases through a systematic approach by researchers with expertise in the subject matter. The diverse nature of the included articles provided insight into the application of relational autonomy in various healthcare disciplines over an extensive period of time (20 years), and across geographical contexts (mainly across the Global North). While such diversity informs a comprehensive overview of the study topic, the inclusion of articles written in English may have resulted in omitting articles that portray varying socio-cultural representations of relational autonomy. Moreover, as solely 2 articles originated from the Global South (Colombia and South Africa), more research is warranted to better understand relational autonomy in pediatric care across this region.

## Conclusion

This scoping review described the nature and scope of literature on relational autonomy in the context of pediatrics. Findings suggest that relational autonomy offers a holistic lens for encapsulating the diverse realities of exercising autonomy in pediatric care for children, caregivers, and healthcare providers. Relational autonomy emerged as a dynamic and complex concept which has been applied to various healthcare disciplines across various geographical contexts. The purpose of relational autonomy was predominantly associated with enhancing understandings of how triadic decision-making processes occur between children, caregivers, and healthcare providers. While relational approaches to care were captured by various concepts and terms, the theoretical underpinnings of relational autonomy were broadly absent from literature. Most importantly, children’s perspectives informing discussions of relational autonomy were also missing.

Future initiatives on this topic could consider strengthening the theoretical underpinnings of relational autonomy to address this theory-practice gap. Moreover, additional research could explore how relational autonomy can be linked to other dimensions of healthcare practice, including in the context of nursing. In pediatrics, relational autonomy research should also reflect the lived realities of children. Emerging knowledge on children’s experiences can reveal how children of varying ages and abilities perceive their developing sense of autonomy in healthcare.

Collectively, these findings shed light on the current state and nature of literature on relational autonomy in pediatrics which can inform the work of a wide range of healthcare providers who care for children and families, as well as managers, administration, policy makers, and researchers. Moreover, findings can add nuance to the development of institutional healthcare policies and guidelines. In particular, incorporating relational autonomy in child- and family-centered care philosophies can enhance understandings of child-caregiver-provider relationships dynamics and strengthen the promotion of children’s rights in pediatrics.^61^

## Data Availability

The dataset generated and analyzed in this review is available upon reasonable request.*
